# Updating social evaluation during sleep

**DOI:** 10.1038/s41539-025-00356-9

**Published:** 2025-08-30

**Authors:** Haoyun Zhao, Xiao Lin, Kai Yuan, Xiaoqing Hu, Xikai Wang, Waxun Su, Qiandong Wang, Lin Lu

**Affiliations:** 1 Peking-Tsinghua Center for Life Sciences and Academy for Advanced Interdisciplinary Studies, Peking University, Beijing, 100871 China; 2 Peking University Sixth Hospital, Peking University Institute of Mental Health, NHC Key Laboratory of Mental Health (Peking University), National Clinical Research Center for Mental Disorders (Peking University Sixth Hospital), Beijing, China; 3 Department of Psychology, The State Key Laboratory of Brain and Cognitive Sciences, The University of Hong Kong, Hong Kong, SAR China; 4 Beijing Key Laboratory of Applied Experimental Psychology, National Demonstration Center for Experimental Psychology Education (Beijing Normal University), Faculty of Psychology, Beijing Normal University, Beijing, China; 5 PKU-IDG/McGovern Institute for Brain Research, Peking University, Beijing, China

**Keywords:** Psychology, Psychology

## Abstract

Sleep is instrumental in the formation of long-lasting memories, including social evaluations and social knowledge. The modification of social evaluations holds profound significance for understanding and shaping societal dynamics. Here, we investigated how sleep could contribute to updating the social evaluation of a person generally perceived as unattractive. We found that, compared with uncued names, auditory cueing (by playing the acoustic name+positive trait pairs) during sleep increased the perceived attractiveness of the mental representations of faces associated with the cued names. Notably, the number of slow oscillations detected during sleep was significantly positively correlated with the attractiveness ratings of the faces corresponding to the cued names. Importantly, a control experiment revealed that mere name exposure without positive traits during sleep did not enhance mental facial representations. These results highlight sleep’s active role in updating social evaluations and suggest that sleep-mediated social evaluation updating can be applied in various social contexts.

## Introduction

Social evaluations—the judgments and impressions we form about others—are deeply rooted in memories that integrate perceptual cues (e.g., facial features) and semantic knowledge (e.g., personality traits)^1,2^. These evaluations are often biased by heuristics such as the attractiveness halo effect, where individuals who are generally perceived as unattractive are systematically perceived as less competent, trustworthy, and likable^3,4^. Previous bias reduction interventions have relied on conscious and effortful strategies, such as counter-stereotype training. However, attitudes often resist deliberate modification due to defensive reactions^5^. A critical challenge, therefore, lies in identifying pathways through which social evaluations can be modified without engaging conscious control mechanisms.

Sleep, particularly slow-wave sleep (SWS), provides a unique neurobiological state for modifying social evaluations because of its role in memory reprocessing and affective integration. During SWS, hippocampal-neocortical interactions based on spindle-oscillation coupling facilitate the selective strengthening and updating of associative memories, including affective valences attached to social stimuli^6–8^. While previous research has shown that sleep learning and memory reactivation can influence subsequent memory representations and behavior^9–11^, it remains unclear whether these processes specifically affect the updating of social evaluations during sleep. Pioneering work by Hu et al. (2015) proposed that targeted memory reactivation (TMR) during sleep could enhance pre-sleep anti-bias training, although subsequent replication attempts have yielded mixed results^12,13^. While these findings remain debated, they highlight the potential application of sleep-mediated interventions for modulating implicit associations. Since social evaluations are rooted in the associative memories that bind individuals with affective attributes, remembering more negative traits or having negative memories of a person’s facial appearance can lead to negative evaluations. In fact, the facial appearances contained in our memories, i.e., mental facial representations, can bias our perception and evaluation and are closely correlated with stigma and prejudice^14,15^. This raises a fundamental question: can sleep be leveraged to reconstruct social evaluations through associative learning?

**Fig. 1:**
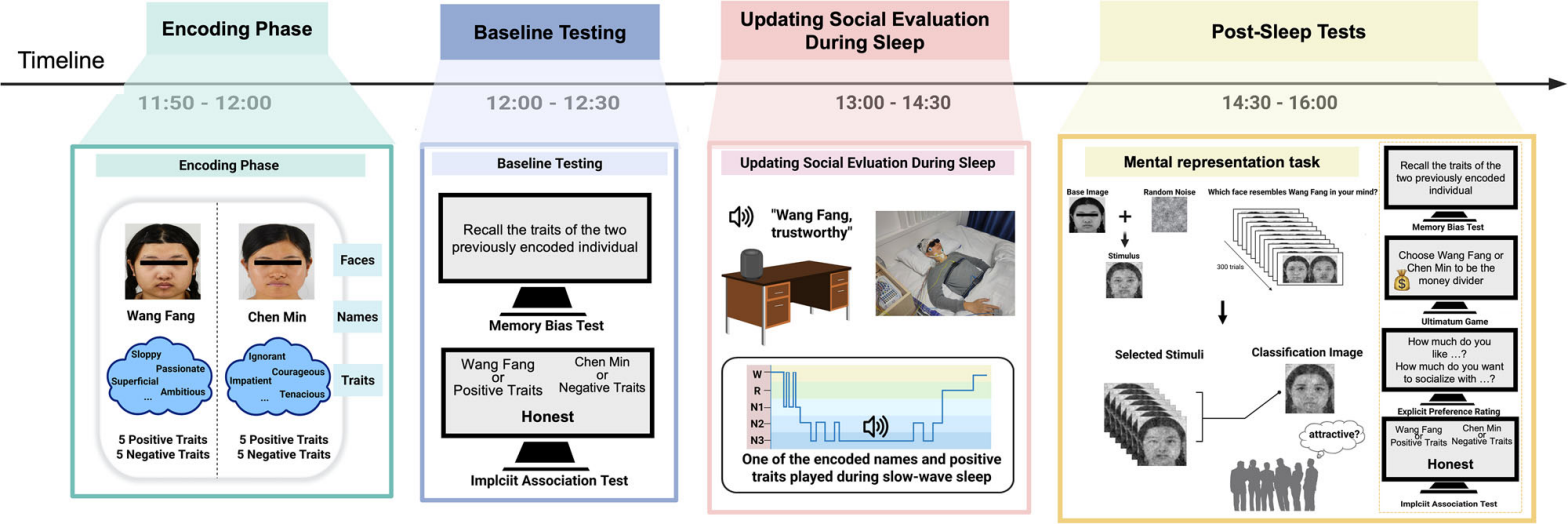
Overview of the study. The study consisted of four different phases. During the encoding phase, participants learned two faces with 10 personality trait descriptions. After encoding the faces and personality traits, the memory of those descriptions was tested, and an adapted implicit attitude test (IAT) was conducted. During SWS sleep, name+positive trait pairs were presented to the sleeping participants. After sleeping, participants performed post-sleeping tests [including mental facial representations, memory bias tests, and implicit (measured by the IAT) and explicit (measured by the ultimatum game and explicit preference rating test) attitudes]. *Note*. All facial images used in this study were obtained from the Tsinghua University Facial Expression Database^36^, and the original database confirms that written informed consent was obtained from all facial models for the use of their images in scientific research, including publications.

Our study aimed to address this gap by investigating whether social evaluations could be improved during sleep by linking acoustically cued names of individuals who were generally perceived as unattractive to novel positive traits during SWS. While modifying social evaluations during wakefulness may be more economical and cost-effective, this process often involves controlled cognitive processes that may conflict with preexisting perceptions or attitudes. For example, conscious efforts to suppress biases can paradoxically reinforce them through ironic rebound effects^5^. In contrast, sleep-mediated updating can bypass conscious resistance and may be more effective than updating participants while they are awake. Indeed, empirical studies have demonstrated that sleep-specific interventions—such as targeted memory reactivation (TMR)—produce unique behavioral effects that are not replicable when applied to participants who are awake^13^. This study tested the hypothesis that sleep-mediated associative learning can counteract attractiveness-based stigma by linking the acoustically cued names of individuals generally perceived as unattractive to novel positive traits during SWS. To assess social evaluations, we focused primarily on facial mental representations and examined social attitudes and the memory of personality traits. On the basis of previous sleep and TMR research, we hypothesized that, upon exposure to acoustic name+positive trait pairs during SWS, participants would form a more favorable mental representation of the cued individual and that their attitudes and memory biases toward that individual would become more positive (an overview of the study is presented in Fig. 1). At a broader level, this research could inform initiatives aiming to diminish stigma and prejudice toward various populations (e.g., mental illness, stigmatized groups, or minorities).

## Results

### Presenting acoustic name+positive trait pairs during sleep improved the attractiveness of mental facial representations

**Fig. 2:**
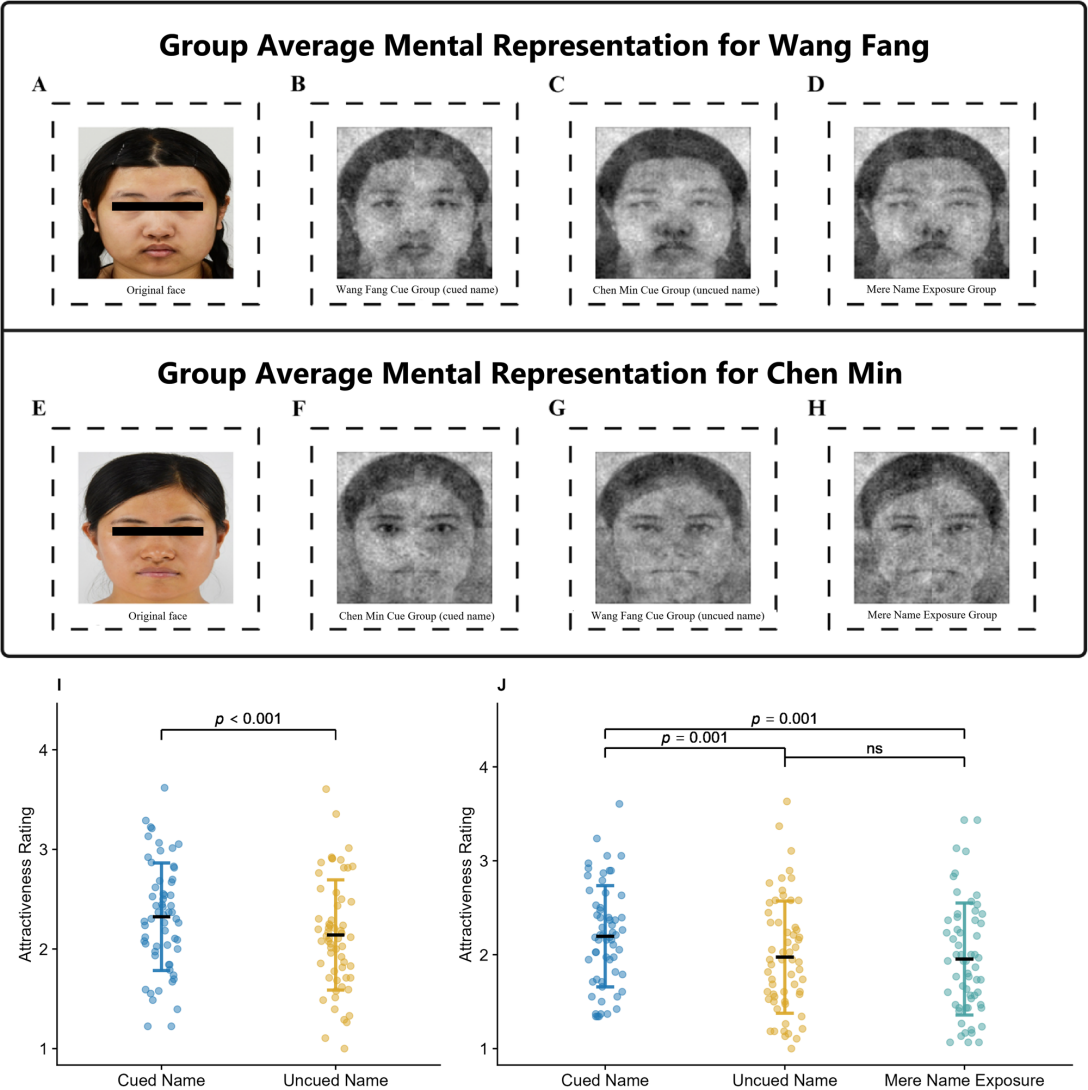
The mental facial representation results. **A**–**D** The original face of Wang Fang, the mental facial representations of the Wang Fang Cue Group (cued name), the Chen Min Cue Group (uncued name), and the Mere Name Exposure Group for Wang Fang’s face. **E**–**H** The original face of Chen Min, the mental facial representations of the Chen Min Cue Group (cued name), the Wang Fang Cue Group (uncued name), and the Mere Name Exposure Group for Chen Min’s face. **I** Attractive rating differences in mental representations between the cued and uncued names. **J** Attractive rating differences in mental representations between the Wang Fang cued name, the Wang Fang uncued name, and the Wang Fang mere name exposure face. *Note*. The images of the faces represented in the figure have been approved for publication.

As shown in Fig. 2I, the Linear Mixed Model (LMM) revealed that the main effect of the condition was significant (*F*(1, 8984) = 107.07, *p* < 0.001), and the attractiveness ratings of the faces associated with the cued names were significantly higher than those associated with the uncued names (*B* = 0.18, *SE* = 0.02, *z* = 10.348, *p* < 0.001). Thus, upon the presentation of acoustic name+positive trait pairs, participants effectively reshaped their mental representation of the individual after exposure to positive traits associated with that individual’s name during sleep.

To justify the use of a single face identity (Wang Fang) in the name-only cuing experiment, we first confirmed that the cuing effects in the main experiment (name+attribute) did not differ between the two faces. A linear mixed-effects model analysis revealed no significant interaction between the cueing condition and face identity (*F*(1, 74) = 0.55, *p* = 0.462), indicating equivalent effects for both face identities. Subsequent analysis for Wang Fang’s face also revealed a significant main effect of the condition (*F*(2, 103) = 7.86, *p* < 0.001). Post-hoc comparisons revealed that the attractiveness ratings of the cued name were significantly higher than those of the uncued name (*B* = 0.22, *SE* = 0.07, *z* = 3.36, *p*_*adjusted*_ = 0.001) and mere name exposure face (*B* = 0.24, *SE* = 0.07, *z* = 3.44, *p*_*adjusted*_ = 0.001). There was no significant difference in ratings between the uncued name face and the face with only name exposure (*B* = 0.02, *SE* = 0.07, *z* = 0.29, *p*_*adjusted*_ = 0.774). The lack of a positive effect from the mere name exposure condition suggests that the positive trait associations related to an individual’s name, rather than the name itself, significantly impact mental facial representations.

To test whether the observed effects were specific to the cued stimuli, we conducted an additional analysis comparing the attractiveness ratings of mental face representations for the uncued name (Chen Min’s face) between the experimental group and the mere name exposure group. This analysis revealed no significant difference in the facial attractiveness ratings for the uncued name between the mere exposure group and the experimental group (*F*(1, 66) = 0.74, *p* = 0.393, see Supplementary Fig. 3).

Furthermore, we examined the potential confounding effects arising from different sleep characteristics across different groups. While most sleep characteristics were matched, there were differences in N1 sleep duration and percentage (see Table 1). However, since auditory cues were delivered primarily during SWS, these N1 differences are unlikely to have influenced the results. This was confirmed by the lack of significant correlations between N1 duration/percentage and attractiveness ratings of the mental representation of the cued name (N1 duration: *r*(104) = −0.047, *p* = 0.632; N1 percentage: *r*(104) = -.096, *p* = 0.330). In addition, we found no statistically significant differences in the average duration of cue played or the average number of cues played among the two cue groups and the mere name exposure group (average duration of cue played: *F* (2,96) = 1.89, *p* = 0.157, η²_p_ = 0.04; average number of cues played: *F* (2,96) = 1.99, *p* = 0.142, η²_p_ = 0.04) (see Supplementary Table 6).

**Table 1:** Demographics and sleep characteristics of the included participants

	Wang Fang Cue Group	Chen Min Cue Group	Mere Name Exposure Group	*F*	*P*-value
Age (mean ± sd, years)	21.84 ± 3.45	21.84 ± 2.51	20.57 ± 1.79	2.35	0.10
Gender					
Female	19	19	14	/	/
Male	19	19	16		
Occupation					
Student	36	38	30	/	/
Office Worker	2	0	0		
Nationality					
Chinese	37	37	30	/	/
Malaysian*	1	1	0		
Ethnicity					
Han	35	35	27	/	/
Ethnic minorities	3	3	3		
N1 Duration (mins)	3.51 ± 3.35	2.75 ± 2.72	1.65 ± 1.56	3.96	0.02
N1 Percentage (%)	5.36 ± 5.35	4.23 ± 4.54	2.58 ± 2.48	3.34	0.04
N2 Duration (mins)	33.82 ± 14.90	33.96 ± 13.51	34.25 ± 16.40	0.01	0.99
N2 Percentage (%)	48.97 ± 17.93	48.11 ± 16.73	51.40 ± 19.61	0.29	0.75
N3 Duration (mins)	29.28 ± 18.64	31.09 ± 17.61	27.58 ± 14.18	0.36	0.70
N3 Percentage (%)	41.03 ± 20.82	42.74 ± 19.74	42.27 ± 20.29	0.07	0.93
REM Duration (mins)	3.73 ± 5.60	3.97 ± 7.00	2.90 ± 3.83	0.31	0.73
REM Percentage (%)	4.67 ± 7.02	4.92 ± 8.04	3.76 ± 4.97	0.25	0.78
Number of SO*	297.11 ± 72.00	299.53 ± 68.35	313.67 ± 71.44	0.52	0.60
Number of Spindles	252.03 ± 146.19	282.68 ± 127.81	290.17 ± 95.58	0.90	0.41
Number of SO-spindle coupling	78.21 ± 49.09	85.68 ± 48.65	86.83 ± 39.12	0.37	0.69
Sleep Efficiency (%)	76.36 ± 20.83	78.03 ± 17.14	73.65 ± 21.21	0.42	0.66
WASO* (mins)	18.26 ± 20.33	14.07 ± 14.77	18.55 ± 18.25	0.71	0.49
Sleep Latency (mins)	4.82 ± 6.79	5.92 ± 6.05	5.03 ± 6.30	0.31	0.73
REM Latency (mins)	60.50 ± 16.00	54.58 ± 19.58	60.27 ± 14.66	0.70	0.50
Total Sleep Time (mins)	70.34 ± 19.16	71.78 ± 16.72	66.38 ± 19.06	0.76	0.47
Total Wake Duration (mins)	19.54 ± 19.09	18.04 ± 14.77	23.58 ± 19.09	0.86	0.43

*native Chinese speaker.

*SO slow oscillation.

*WASO wakefulness after sleep onset.

**Fig. 3:**
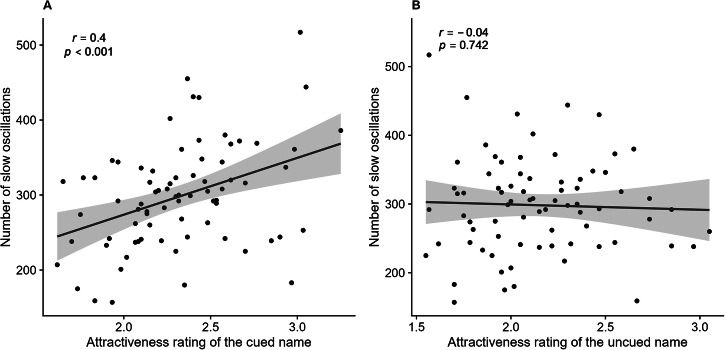
The correlations between the number of slow oscillations and the perceived attractiveness of the mental facial representations. **A** The correlation between the number of slow oscillations and the attractiveness rating of the faces associated with the cued names. **B** The correlation between the number of slow oscillations and the attractiveness rating of the faces associated with the uncued names.

Finally, we examined how the perceived attractiveness of mental facial representations was related to the number of SOs during sleep. We assessed the Person correlation between the number of SOs and the attractiveness ratings toward the cued and uncued names, respectively. The findings, presented in Fig. 3 (also see Supplementary Fig. 4 for a specific depiction of Wang Fang’s face), revealed a significant positive correlation between the number of SOs and the attractiveness ratings of the faces associated with the cued names (*r*(74) = 0.40, *p*_*adjusted*_ < 0.001). In contrast, the correlation between the number of SOs and attractiveness ratings for the faces associated with the uncued names was not significant (*r*(74) = −0.04, *p*_*adjusted*_ = 0.742).

### Presenting acoustic name+positive trait pairs during sleep improved the memories of the positive personality traits of the individuals with cued names

**Fig. 4:**
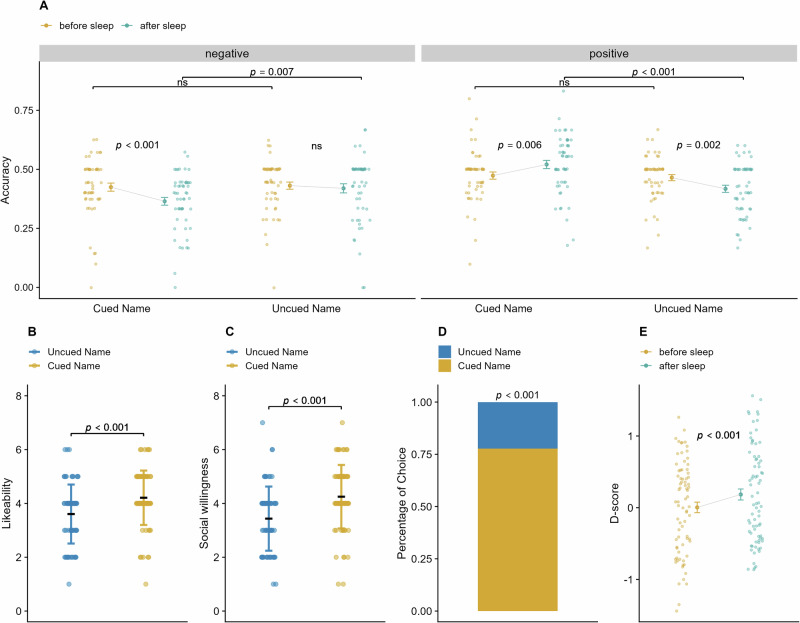
Memory of the personality traits and attitudes results. **A** Recall accuracy of the personality traits of individuals with cued and uncued names before and after cue exposure during sleep. **B**–**D** Explicit measures of likeability, social willingness to engage, and social trust toward the individual with cued and uncued names. **E** Implicit attitudes (measured by implicit association tests, IAT) toward the individuals with cued and uncued names in pre- and post-sleep. A higher D-score represents a more positive implicit attitude toward the individual with the cued name than toward the individual with the uncued name.

We next sought to explore whether our manipulation produced changes in the recall of positive or negative personality traits of the two faces. The means (*SDs*) of memory bias for positive and negative traits of the cued and uncued names before and after sleep are shown in Table 2. We conducted a repeated measures ANOVA (2 × 2 × 2) to analyze the participants’ memory biases. This analysis took into account within-subject factors, including the facial type (associated with the cued and uncued names), time (before and after sleep), and valence of the traits (positive and negative). The results are shown in Fig. 4A. We found that the main effect of valence was significant, *F*(1,53) = 17.43, *p* < 0.001, η²_p_ = 0.25, and positive traits were better remembered than negative traits were (mean ± *SD*; positive traits = 0.47 ± 0.11, negative traits = 0.41 ± 0.13). We also found a two-way interaction effect between time and valence, *F*(1,53) = 4.46, *p* = 0.039, η²_p_ = 0.08, and between face type and valence, *F*(1,53) = 9.66, *p* = 0.003, η²_p_ = 0.15. Furthermore, we detected a significant three-way interaction effect among time, valence, and face type, *F*(1,53) = 15.00, *p* < 0.001, η²_p_ = 0.22. There were no other significant effects, all *p*s > 0.05. Further simple effect analyses revealed that before sleep, there were no significant differences in the memories for positive and negative traits of the two face types (positive traits: *t*(53) = 0.52, *p* = 0.606, Cohen’s d = 0.06; negative traits: *t*(53) = −0.33, *p* = 0.741, Cohen’s d = −0.04). However, after sleeping, participants remembered more positive traits (*t*(53) = 4.54, *p* < 0.001, Cohen’s d = 0.68) and fewer negative traits (*t*(53) = −2.81, *p* = 0.007, Cohen’s d = −0.36) for the cued name than for the uncued name. Additionally, after sleep, participants showed a significant increase in memories of positive traits and a significant decrease in memories for negative traits for the cued name, compared with pre-sleep levels (positive traits: *t*(53) = 2.88, *p* = 0.006, Cohen’s d = 0.31; negative traits: *t*(53) = −3.52, *p* < 0.001, Cohen’s d = −0.39). Conversely, for the uncued name, a significant decrease in memories of positive traits and no significant change in memories of negative traits were found (positive traits: *t*(53) = −3.29, *p* = 0.002, Cohen’s d = −0.31; negative traits: *t*(53) = −0.54, *p* = 0.589, Cohen’s d = −0.07). Additionally, none of the participants recalled any of the positive traits presented during sleep, as indicated by zero reports of the traits played during sleep in the memory test. This suggests that the cues were processed unconsciously without entering conscious awareness, supporting the interpretation that the effects observed were driven by unconscious processing during sleep. These results confirmed our hypothesis that name+positive trait pair exposure during sleep biased the memories of the participants, leading them to remember more of the positive traits of the cued name, even if those traits were not played during sleep.

**Table 2:** Memory bias for positive and negative traits of the individuals with cued and uncued names before and after sleep

	Before sleep		After sleep	
	Individuals with cued name	Individuals with uncued name	Individuals with cued name	Individuals with uncued name
Memory for positive traits (mean ± *SD*)	0.47 ± 0.11	0.46 ± 0.10	0.52 ± 0.13	0.42 ± 0.11
Memory for negative traits (mean ± *SD*)	0.42 ± 0.13	0.43 ± 0.11	0.36 ± 0.12	0.42 ± 0.14

### Presenting acoustic name+positive trait pairs during sleep improved the implicit attitudes toward the individuals with cued name

We employed the implicit association test (IAT) to assess the implicit attitudes of the participants toward the cued and uncued names before and after acoustic cue exposure during sleep. As shown in Fig. 4E, we found a significantly improved implicit attitude toward the cued name compared with the uncued name after sleep (mean ± *SD*; before sleep: 0.00 ± 0.64; after sleep: 0.18 ± 0.67; *t*(75) = 3.50, *p* < 0.001, Cohen’s d = 0.40). These findings suggest that exposure to the acoustic name+positive trait pairs during sleep could improve the implicit social attitudes. While the significant improvement in IAT scores suggests that participants may have changed their implicit social evaluations, the precise nature of these changes (whether reflecting new semantic associations or adjustments to existing associations) requires further investigation.

### Presenting acoustic name+positive trait pairs during sleep improved the explicit attitudes toward the individuals with cued name

In addition to the implicit attitude measures, participants gave their explicit evaluations after sleeping. The results, as shown in Fig. 4B–D, revealed that participants showed a significantly greater degree of likeability and social willingness to engage with the individuals with cued names than those with uncued names (degree of likeability: mean ± *SD*; cued name: 4.21 ± 1.01; uncued name: 3.61 ± 1.10; *t*(75) = 3.55, *p* < 0.001, Cohen’s d = 0.41; degree of social willingness: mean ± SD; cued name: 4.25 ± 1.18; uncued name: 3.43 ± 1.19; *t*(75) = 4.36, *p* < 0.001, Cohen’s d = 0.50). In addition, we measured participants’ social trust by asking them to choose which person they would like to be in charge of deciding how to divide the money while playing an ultimatum game. The results revealed that the choice of selecting the individuals with the cued name (77.8%) was significantly greater than that with an uncued name (22.2%), Χ^2^(1) = 16.67, *p* < 0.001. These findings suggest that the semantic association exposure during sleep not only improves the implicit attitudes but also enhances the explicit social attitudes.

## Discussion

By playing acoustic name+positive trait pairs during sleep, we showed that the social evaluation of a specific person can be unconsciously updated during sleep through the formation of semantic associations. This reshaping was multifaceted, as evidenced by improved mental representations, the ability to recall more positive personality traits, and the demonstration of more positive attitudes (both explicit and implicit) toward the individuals with cued names than toward those with uncued names. While previous studies have shown that humans can learn new information during sleep^16–18^, we provide new evidence that social evaluation can be reshaped during sleep through aurally played, name-trait semantic associations.

Our findings present two perspectives for future studies. First, previous studies have focused mainly on changing people’s attitudes and cognition during wakefulness^19–21^, whereas our research has examined the role of sleep in this process. Our study demonstrated that name+positive trait pair exposure during sleep can modulate social evaluations in a controlled laboratory setting. Second, we demonstrated that we could improve social evaluations of faces generally perceived as unattractive. This opens possibilities for changing people’s stigma and attitudes toward disadvantaged groups and reducing the stigma associated with such groups, such as those with mental illness, by reshaping the social evaluation.

First, we found that the mental facial representation of faces that were generally perceived as unattractive became more attractive after name+positive trait pair exposure during sleep. These results are consistent with two non-mutually exclusive interpretations. This could be due to the sleep-mediated integration of novel positive traits (i.e., those that were not explicitly learned pre-sleep) into existing memories. Alternatively, sleep-dependent strengthening of pre-sleep name-trait associations may also have contributed to these results, even though these associations were not directly reactivated during sleep. While the lack of overlap between pre-sleep learned traits and sleep-played traits indirectly supports the new associative learning and integration account, our design cannot rule out alternative reactivation and consolidation accounts. Our study—although not designed to resolve this debate—highlights the need for more targeted designs to dissociate memory consolidation from new learning. While our findings highlight promising avenues for reducing social biases, they equally underscore the need for proactive neuroethical guidelines. Explicit acknowledgment that sleep-based interventions could be exploited for unethical purposes (e.g., political propaganda or commercial manipulation) is critical. The same mechanisms that could improve intergroup attitudes might theoretically be exploited for covert influence, necessitating careful consideration of consent protocols and application boundaries. We echo the concerns raised in recent neuroethical discussions^22^ and call for the development of regulatory frameworks to ensure the responsible use of this technology.

Several studies have shown that name exposure can increase an individual’s familiarity with and preference for a cued item^23^. For example, presenting the name of a familiar snack item acoustically during a nap significantly improved the participant’s preference for that item relative to items not externally cued^24^. To isolate the effect of name exposure, we included a control group that was exposed only to the name during sleep. However, we did not find improved mental representations in this control group. One possible explanation is that the previous study used familiar and highly valued snack items (i.e., participants’ favorite snacks), whereas in our study, we deliberately chose faces with low value (i.e., faces generally perceived as unattractive). We did this for two reasons: first, if the face itself is attractive and likable, the improvement effect may be too subtle to detect; second, we wanted our study to inform and inspire future studies, especially those aimed at reducing the stigma toward individuals with negative social labels. One interpretation of the inconsistency is that mere name exposure has an effect when the stimuli are not of low value.

Notably, while the “mere name exposure” control condition ruled out the possibility that name exposure alone drove changes in social evaluations, our study did not directly contrast the effects of pairing names with positive traits with pairing names with neutral or negative traits. The lack of a name + neutral trait control condition might confound the impact of sound duration on the results. Additionally, the lack of a name + negative trait control condition limits our ability to conclude that the observed effects are uniquely tied to positive valence, although such designs require careful ethical consideration. Future studies should compare valence-specific associations to determine whether sleep allows bidirectional social evaluation updates.

Second, we found that acoustic name+positive trait pair exposure during sleep significantly biased the memory of traits. We did not directly test the participants’ memories for the exposed cues; instead, we tested for words that shared similar attributes with the exposed traits (i.e., the learned positive traits before sleep differed from the positive traits being played during sleep). These results were consistent with the research reporting that participants were more likely to remember other positive traits about a target after being exposed to positive words, even when the exposure was unconscious^25,26^. Moreover, a recent study revealed that the reactivation of individual memories during sleep evoked reinstatement of their context, thereby impacting the consolidation of associated knowledge^27^. Our results provide complementary evidence that being exposed to positive traits increases subsequent memories of traits belonging to the same categories.

Finally, we measured both explicit and implicit attitudes and found more positive attitudes toward the person whose name was associated with positive traits during sleep. These results echo those of a previous study that reported reduced implicit social biases after sleep, which was attributed to the reactivation of prior training while awake^28^. In contrast, our study did not involve wakeful training, and our manipulation occurred entirely during sleep. Thus, these results further confirmed for the possibility of acquiring new information during sleep^29^, which goes beyond the well-documented role of sleep in consolidating previously acquired memories. The sleeping brain undergoes neural activity that supports the processing of information^30,31^, which may explain why being exposed to name+positive trait pairs during sleep positively impacts attitudes.

This study has several limitations that may constrain our interpretations. First, we used polysomnography (PSG) instead of high-density electroencephalography (EEG) to monitor sleep, making the examination of cue-elicited EEG activity changes challenging. Relatedly, it remains unclear whether the exposure effect is contingent upon the SO upstate or downstate, as numerous studies emphasize that the benefits could be associated with the SO upstate^7,32,33^. Second, given that we did not include a follow-up test, whether this reshaping of the social evaluation is long-lasting warrants future investigation. Third, we did not include a wakefulness control condition; future work could directly compare the efficacy and cost-effectiveness of sleep-mediated updating versus wakeful updating.

Finally, the generalizability of our findings may also be constrained by the specific characteristics of our study population and materials. Our study recruited young Chinese adults. Cultural and age-related differences in social perception, such as varying attractiveness norms, might influence the observed effects. Furthermore, our stimuli consisted exclusively of unattractive female faces. It is unclear whether similar effects would be observed for male faces or faces with different baseline attractiveness levels. Additionally, our study focused only on the attractiveness dimension. Future research could explore other dimensions, such as social status or trustworthiness, which may also play important roles in social evaluation. We encourage subsequent studies to involve diverse populations and materials and examine cross-cultural applicability to increase the generalizability of the findings.

In conclusion, this study offers valuable insights into how sleep influences social cognition, providing a new approach to improving people’s social evaluation. However, more research is needed to fully understand the neural mechanisms employed, the long-term effects of this influence, and the potential practical applications. By building on these studies, we can endeavor to foster more harmonious interpersonal relationships and enhance understanding among diverse social groups, which may contribute to a reduction in prejudice and discrimination.

## Methods

### Participants

A total of 138 healthy adults (73 females; age: *M* ± *SD*, 21.69 ± 2.75 years old; age range: 18–34 years old) recruited from the local university community were enrolled in the study. The ethics review committee of Beijing Normal University approved the study (IRB number: BNU202309280139). All participants provided written informed consent and received monetary compensation.

The sample size was determined through a multifaceted approach to ensure methodological rigor. First, studies in this field^13^ typically involved 15–30 participants per experimental condition. Second, an a priori power analysis was conducted via G*Power software^34^ (version 3.1) for within-subject paired-sample *t*-tests. With α = 0.05 (two-tailed), a medium-sized effect (Cohen’s d = 0.66; based on a previously published work^35^), and 80% power, the analysis indicated a minimum sample size of *N* = 21 participants. In addition, we intentionally recruited participants beyond this minimum target to accommodate anticipated exclusions (e.g., slow wave sleep duration < 5 minutes or reported awareness of auditory cues during sleep) and to increase the robustness and reliability of our results. The participants were native Chinese speakers with normal vision and hearing and had no reported history of neurological, psychiatric, or sleep disorders. Furthermore, individuals who self-reported an inability to recognize faces correctly were excluded from study recruitment.

We enrolled participants in two separate batches. The participants were randomly assigned to one of two groups in the first batch, with the cued face being named either “Wang Fang (王芳)” or “Chen Min(陈敏)”. Specifically, if a participant was assigned to the Wang Fang Cue Group, “Wang Fang” was the cued name and “Chen Min” was the uncued name, and vice versa (see Table 1). These participants were exposed to acoustic name+positive trait pairs during SWS (Wang Fang Cue Group: age: M ± SD, 21.84 ± 3.45 years; age range:18–34 years; Chen Min Cue Group: age: *M* ± *SD*, 21.84 ± 2.51; age range: 19–29 years). To exclude the possibility that the presentation of name alone could improve mental facial representations, we recruited another batch of participants who were only re-exposed to the spoken names during SWS (Mere Name Exposure Group: age: *M* ± *SD*, 20.56 ± 1.79 years; age range: 18–24 years).” Participants from different groups were matched for key demographic variables (Table 1).

Initially, 270 individuals were assessed for eligibility, of whom 132 were excluded due to non-nocturnal sleeping habits (*n* = 35), self-reported sleep disturbances (*n* = 27), sensitivity to the sleeping environment (*n* = 56), psychological disorders (*n* = 5), being psychology majors (*n* = 6), or declining to participate (*n* = 3). A total of 138 participants were enrolled in the study. For data analysis, we excluded data from participants who had a slow-wave sleep duration of less than 5 minutes (*n* = 28) or who reported hearing the sound cues during sleep (*n* = 4). Thus, data from 106 participants were analyzed. Participants from the two separate batches were matched for key demographic factors. The demographic characteristics of the included participants are presented in Table 1.

### Materials

We selected 31 color female facial images with neutral facial expressions to use as facial stimuli from the Tsinghua University Facial Expression Database^36^. The original database explicitly states that all facial models provide written informed consent for their photographs to be used in scientific research, including experiments, publications, and presentations^36^. Since our study strictly adheres to the database’s usage guidelines, no additional consent was required for the publication of these images. To obtain attractiveness ratings, we recruited an additional 56 participants from the Naodao online platform (https://www.naodao.com/) to rate the attractiveness of faces on a scale of 1 (extremely low attractiveness) to 5 (extremely high attractiveness) (see Supplementary Fig. 1). On the basis of the average attractiveness ratings, we selected two facial images rated as least attractive for our test stimuli (see Supplementary Note 1). We selected the attractive dimension because attractiveness is a core trait of faces that can significantly influence social evaluations and judgments^3,4^. We chose to use a single gender (female) to control for potential confounding variables related to gender, ensuring that any observed effects were due primarily to the experimental manipulation rather than differences between male and female faces.

The facial stimuli used in the reverse correlation image classification (RCIC) task, which was used to obtain the mental facial representations, were subjected to the image processing techniques used in prior research^2,37,38^ (see Supplementary Note 2 and Supplementary Fig. 2). All facial stimuli used in the RCIC task were converted to grayscale, which is a requirement of the RCIC technique. The pixel dimensions of the two facial images used in the encoding phase were both standardized to 512 × 512 pixels. Each of the two facial images was morphed separately with another female face sourced from the Tsinghua University Facial Expression Database^36^. The morphed images were then subjected to a blurring effect. Positive and negative noise was added to the blurred images. The images were paired: one with positive noise and the other with negative noise, resulting in 300 image pairs for each of the two potential cued face targets.

The names used in the study were representative of those commonly used in the Chinese population. The surnames were chosen from the top 60 most common surnames in China, as documented in the classic text “Hundred Family Surnames”. The given names were selected on the basis of popularity and cultural relevance, as indicated by authoritative sources such as Xinhua Net and the National Bureau of Statistics of China. Once both sets of names (surnames and given names) were compiled, they were paired in a systematic manner to form full names. The pairing was performed by either a random combination or following a pattern reflecting common naming practices in China. The goal was to generate a pool of names that would be typical, realistic, and recognizable to native Chinese individuals.

We recruited 50 native Chinese speakers from the Naodao online testing platform to rate the names for commonness (1 for uncommon, 2 for common), ordinariness (1 for absurd, 2 for ordinary), and gender association (1 for male, 2 for female). The order in which these names were presented to participants was randomized to prevent bias. On the basis of this survey, two female names, “Wang Fang” and “Chen Min”, both of which received the highest average ratings in commonness and ordinariness (both had an average rating of 2 in each category), were selected as test stimuli.

The personality traits employed in the encoding phase of the study were derived from Zhang et al., (2018)^39^, in which 160 Chinese personality trait words, encompassing both positive and negative connotations, were evaluated by independent raters on the basis of valence (*n* = 66; mean ± *SD*; positive traits = 5.60 ± 0.25; negative traits = 2.40 ± 0.35), arousal (*n* = 66; mean ± *SD*; positive traits = 4.71 ± 0.36; negative traits = 4.74 ± 0.34), familiarity (*n* = 64; mean ± *SD*; positive traits = 6.05 ± 0.34; negative traits = 6.03 ± 0.33), and meaningfulness (*n* = 60; mean ± *SD*; positive traits = 5.98 ± 0.28; negative traits = 6.02 ± 0.21). We selected 20 personality traits from this comprehensive set, allocating ten distinct traits to each of the face-name pairs, ensuring an equal balance of five positive and five negative traits (for details, see Supplementary Note 3, Supplementary Tables 1 and 2).

Additionally, the attribute words employed in the implicit association task (IAT), which was used to measure implicit social attitudes, were sourced from a prior study^40^ and were selected to ensure that they did not overlap with the personality traits used in the encoding phase (for details on attribute and concept words used in the IAT task, refer to Supplementary Table 3).

During sleep, participants were presented with the name of the cued face paired with 16 different positive traits (e.g., “Wang Fang, trustworthy” or “Chen Min, trustworthy”). These 16 positive traits, selected from Zhang et al. (2018)^39^, were rated highest in terms of familiarity by 64 independent raters (see Supplementary Table 4). The participants in the Mere Name Exposure Group were presented with only the cued name (e.g., “Wang Fang”). All sounds of the 16 positive traits were generated via an AI voice-over software with uniform parameters (for details, see Supplementary Note 4).

### Procedure

The participants arrived at the sleep monitoring laboratory of Peking University Sixth Hospital at noon, where they provided informed consent. All of the participants were slightly sleep-deprived. They were instructed to maintain their regular sleep schedules on the nights before the experiment. However, to facilitate their ability to fall asleep easily and maintain a longer nap at noon, we woke them up at 6:00 AM; this approach was based on a previous study^41^. Our study consisted of four phases: the encoding phase, baseline testing phase, reshaping social evaluation during sleep phase, and post-sleep testing phase (Fig. 1).

To assess whether mere exposure to the name during SWS could independently enhance mental facial representations, we recruited a third group of individuals who were presented with only the name (“Wang Fang”), without associating it with any positive traits.

### Encoding phase

During the encoding phase of the study (Fig. 1), participants memorized the faces of two individuals, each associated with a distinct name (“Wang Fang” and “Chen Min”), and a set of ten personality traits encompassing five positive and five negative attributes (see Supplementary Table 1). Each participant was given 6 minutes for this memorization task. An AI-generated voice-over was used to acoustically pronounce each name, facilitating the association between the faces, names, and their corresponding traits.

### Baseline (pre-sleep) testing phase

After learning the face-name-trait pairings, participants completed two behavioral tests in the baseline testing session (Fig. 1): the memory bias test, where they freely recalled personality traits associated with two faces, and an implicit association test (IAT). This adapted IAT evaluated participants’ implicit attitudes toward these faces by asking them to categorize names and related positive/negative traits quickly.

Following the encoding phase, participants freely recalled the personality traits of the two individuals presented in the encoding phase. This test required participants to correctly recall as many personality traits as possible for both individuals. The participants’ recollections of the positive and negative traits were collected through an online questionnaire on the Naodao testing platform. Synonyms were not accepted as correct. We used a scoring system where each correctly recalled trait was given a point, and the total score was calculated separately for positive and negative traits. This task was not time-restricted, and participants submitted their responses upon completion. Participants completed this same memory bias test both before and after sleep.

After completing the memory bias test, participants proceeded to the implicit association test (IAT), which measures an individual’s implicit attitudes toward certain concepts or groups^42,43^. In the IAT for this study, participants were required to quickly match two sets of names (e.g., “Wang Fang” and “Chen Min”) with two sets of attribute words (e.g., positive attributes and negative attributes) (see Supplementary Table 5). The results, which were based on the speed of keypress matches, revealed the participants’ potential implicit attitudes toward the two presented individuals. The detailed process can be found in Supplementary Note 5. Each participant underwent the same IAT before and after sleep.

### Acoustic cue exposure during sleep

In the phase of reshaping social evaluation during sleep (Fig. 1), participants were exposed to acoustic cues (name+positive trait pairs) during their SWS phase during a 90-minute nap. Acoustic cues presented to participants during sleep were delivered through an external Bluetooth speaker. Participants were exposed to the name of one encoded individual (either “Wang Fang” or “Chen Min”) paired with 16 different positive words including “confident”, “sincere”, “sociable”, “committed”, “humble”, “patient”, “hopeful”, “reliable”, “fair”, “calm”, “diligent”, “humorous”, “proactive”, “intelligent”, “attractive”, and “honest”. Importantly, only one of the two encoded individuals was associated with positive traits (cued name). The other individual functioned as a control, with the name not being presented to the participants or associated with any words (uncued name). Half of the participants were presented with “Wang Fang” as the cued name, designated as the Wang Fang Cue Group, whereas the remaining participants constituted the Chen Min Cue Group. Each cue (name+positive trait) was repeated 10 times before the next cue was presented.

The duration of each pair of words ranged from 1933 ms to 2125 ms. Additionally, there was a 2-second interval between two consecutive pairs. With the ambient environmental noise level maintained at 45 decibels, the volume of the acoustic cues was set to 60 decibels. This ensured that the sound level reaching the participants’ bedside was approximately 50 decibels (for more details, see Supplementary Note 6). In the event of a sudden change in a participant’s sleep phase, the experimenter would immediately halt the playback and resume once the participant’s sleep phase had stabilized. Cues were presented primarily during slow-wave sleep (SWS/N3), as this stage is optimal for memory reactivation and consolidation processes. We monitored sleep stages continuously via polysomnography to ensure accurate identification of SWS. When we detected that participants were outside of SWS, we paused the cue presentation until SWS was re-established. The cues were only resumed once the participant re-entered SWS. We took several measures to prevent cues from being played during wakefulness. The sleep environment was carefully controlled, and participants were monitored continuously. Additionally, cues were presented at a low volume (50 dB) to reduce the likelihood of arousal. Our post-sleep questionnaires indicated that participants did not recall hearing any cues during wakefulness, suggesting that cue presentation during wakefulness was not a significant factor in our study.

Participants in the mere exposure control group were exposed to the name of one pre-learned face (Wang Fang) during SWS without associated positive traits. The name of the uncued face in this group (Chen Min) was not presented to participants. All other experimental parameters (e.g., sound volume) matched those of the experimental group. The total exposure duration was equivalent for the mere name exposure and the name+positive trait exposure conditions (for details, see Supplementary Table 6.

The acoustic cues were played via the Psychtoolbox^44^ in MATLAB 2020b, ensuring precise timing and synchronization with the polysomnography (PSG) recordings. The playback of these cues was closely monitored, and we recorded the duration and number of cues played (for further details, see Supplementary Note 7).

### Sleep Period and Polysomnographic Recordings

The sleep period began at approximately 1:00 p.m. and lasted ∼1.5 hours (*M* ± *SD* = 69.74 mins ± 18.25 mins). Participants were left to sleep in a laboratory bedroom, and their brain activity was monitored via polysomnography (PSG).

A Compumedics Garel PSG monitoring system (Compumedics Sleep Study System, Melbourne) was used to monitor participants’ sleep. EEG scalp electrodes were attached according to the international 10–20 system at six scalp locations—frontal (F3 and F4), central (C3 and C4), and occipital (O1 and O2)—with each referenced to electrodes on the contralateral mastoid (M1 and M2). A ground electrode was attached to the forehead. In addition, an electrooculogram (EOG), a chin electromyogram (EMG), and a leg EMG were also attached. All electrodes had an impedance of <10 kΩ and were unfiltered and digitally sampled at 256 or 512 Hz. Sleep stage analysis was performed manually, with each epoch lasting 30 seconds. The sleep stages were identified in real-time and scored offline by a trained specialist per the American Academy of Sleep Medicine guidelines. The sleep stages, including NREM and REM, were described by their characteristic PSG patterns and physiological features.

### Post sleep tests

In the post-sleep testing session (Fig. 1), the same participants repeated the same memory bias test and IAT used in the baseline testing session. Subsequently, they completed an ultimatum game to evaluate their levels of social trust toward the two individuals (Wang Fang and Chen Min), a reverse correlation image classification technique (RCIC) task^45^ to assess participants’ mental facial images of those two faces, and an explicit preference rating test to examine participants’ likeability and social interaction willingness toward those two individuals.

The ultimatum game assessed whether acoustic cue exposure during sleep could increase participants’ social trust in the cued individual. After sleeping, the same participants were presented with a scenario involving a hypothetical 500 yuan monetary reward in a single trial. They had to choose either Wang Fang or Chen Min, individuals they had learned about in the pre-sleep phase, to distribute this amount. Participants were told that the selected individual would determine the portion of the virtual reward to be returned to the participant, which would impact their actual compensation: higher virtual rewards would translate to greater actual compensation. Their single-trial decisions were recorded via an online questionnaire on the Naodao testing platform.

To address our central hypothesis regarding the updating of mental facial representations following exposure to name+positive trait pairs during sleep, we obtained each participant’s mental representations of the two learned faces via the RCIC technique^45^, as shown in the right panel of Fig. 1. In each trial, participants were presented with a pair of side-by-side images and tasked with selecting the image that more closely resembled one of two previously memorized individuals, either Wang Fang or Chen Min, via designated key presses. The paired images were generated by adding random sinusoidal white noise to the same base face image, generating different appearances. The test consisted of two rounds, each containing 300 trials, in which participants judged the resemblance to either Wang Fang or Chen Min. Each participant’s individual-level mental representations were generated by averaging the noise patterns from the selected stimuli and overlaying these patterns onto the original base image. The underlying assumption behind this method was that participants would choose the stimulus with the noise that modified the appearance to fit their mental representation. For group-level mental representations, we averaged the noise patterns from these individual mental representations in a group and applied this composite noise to the base image. The group-level mental representations were only used to present the results, not for statistical analysis. Each participant’s individual-level mental representations were assessed online by an independent group that did not include the main experiment participants via the Naodao online testing platform. These raters evaluated the attractiveness of the mental representations on a scale ranging from 1 (extremely low attractiveness) to 5 (extremely high attractiveness).

The explicit preference rating test was designed to determine whether acoustic cue exposure during sleep could enhance participants’ explicit evaluation of the cued person. This test examined participants’ degree of likability and willingness to engage with the individual socially. Using the Naodao online platform, participants completed a questionnaire in which they rated the likeability of Wang Fang and Chen Min on a 1 to 7 scale, with 1 representing extremely low likeability and 7 representing extremely high likeability. They also assessed their willingness to engage socially with both individuals, using the same 1 to 7 scale to indicate their willingness.

### Data analysis

The MATLAB FieldTrip toolbox^46^ was applied to identify the number of slow oscillations (SOs), spindles, and slow oscillation-spindle (SO-spindle) coupling during NREM sleep. We implemented a series of preprocessing steps to ensure data quality and reliability. First, we downsampled the PSG recordings to a frequency of 256 Hz to ensure consistency across datasets. We subsequently performed re-referencing using the M2 channel as the reference. To reduce extraneous noise, we applied a [0.05, 45 Hz] bandpass filter, attenuating high-frequency noise and slow drifts. Additionally, we utilized a discrete Fourier transform (DFT) to specifically remove line noise, thus purifying the signal for further processing. Moreover, we applied the visual artifact rejection algorithm to reduce the remaining artifacts stemming from eye blinks, eye movements, and body movements.

The detection and analysis of slow oscillations and spindles were performed exclusively on EEG segments identified as the N2 and N3 sleep stages (collectively NREM sleep) according to standard AASM criteria. Both the SO and spindle signals were subjected to normalization through z-score transformation. The detection of SOs was facilitated by further filtering the input signal within a [0.25, 1 Hz] frequency band, which corresponded to the typical frequency range of SOs^47^. Negative peaks exceeding 1.5 × SD of the z-scored signal were identified as SO candidates, with duration constraints of 0.8–2 s. Each SO required a preceding positive peak >0.5 ×SD within 1 s before the negative peak. For the detection of sleep spindles, the same filtering approach was applied to the 12–16 Hz band, with an amplitude >1.25 × SD of the z-scored envelope and a duration of 0.5–3 s. Transient bursts were excluded because oscillatory continuity was required. SO-spindle coupling was quantified by aligning the spindle peaks (±1.75 s window) to the SO troughs. The SO phase was extracted via the Hilbert transform, and spindles were assigned to 18 phase bins (20° resolution). Only spindles with peaks occurring ±0.5 s around SO troughs were counted as coupled events. The codes used in the present study were provided online (https://osf.io/8nhv6/).

To better describe the sleep characteristics of the included participants, we also compared the following sleep parameters across different groups (Wang Fang Cue Group, Chen Min Cue Group, and Mere Name Exposure Group): total sleep time, total wake duration, sleep onset latency, durations and percentages of NREM stages 1, 2, and 3, REM sleep duration and percentage, wakefulness after sleep onset (WASO), sleep efficiency (SE), sleep latency (SL), and REM onset latency. These results are shown in Table 1. Notably, we encountered a data collection error with one participant’s sleep data that occurred toward the end of the recording period. Consequently, we utilized the portion of the data that was successfully recorded, which encompasses the majority of the sleep data for this participant.

We first quantified the mental representations of the two learned faces for each participant. An independent sample (*N* = 60, 29 females, age=26.42 ± 6.68 years) was recruited to evaluate the attractiveness of those mental facial representations. The number of independent raters (*N* = 60) for the attractiveness assessment was informed by previous work^48^, which employed a comparable sample size for similar assessments. All individual-level mental representations generated by participants, including those from the Wang Fang Cue Group, Chen Min Cue Group, and Mere Name Exposure Group, were rated on attractiveness by the same 60 independent online raters who did not participate in the main experiment.

To evaluate differences in the perceived attractiveness of mental representations, we conducted a linear mixed-effects model (LMM) with the following Wilkinson notation: rate ~ Condition + (1|subID) + (1|expID). Participant’s ID for the main experiment (expID) was included as a random intercept to account for individual variability in representation generation, whereas the participant’s ID for the ratings (subID) was included as a random intercept to account for individual variability in ratings. The variable ‘Condition’ refers to different conditions, including the cued name (acoustic name+positive trait pairs were presented) and the uncued name (no cues were presented). Additionally, to investigate whether mere exposure to acoustic names during sleep could affect the mental representations, we set up the same model with the conditions that included the cued name, the uncued name, and the mere name exposure face (only acoustic names were presented). Notably, prior to setting up this model, we confirmed via a separate model — rate ~ condition*face names + (1|subID) + (1|expID) — that the cueing effects observed in the main experiment (names + attributes) did not differ between the two faces (see Results section for details). Given that only the name “Wang Fang” was presented in the Mere Name Exposure Group, our comparisons were restricted to the differences in the mental representation of Wang Fang’s face.

LMM was performed via the “lme4” package in R^49^, with *p*-values approximated by the Satterthwaite method (“lmerTest” package^50^). Post hoc pairwise comparisons were conducted with the “emmeans” package^51^, applying the Benjamini-Hochberg false discovery rate (FDR) correction for multiple comparisons.

Memory accuracies were first calculated for pre- and post-sleep conditions (see Supplementary Note 8). These data were then analyzed via a 2 (face type: cued name and uncued name) × 2 (valence of traits: positive and negative) × 2 (time: pre- vs. post-sleep) repeated measures ANOVA to examine any interactions and main effects. In addition, we conducted simple effect analyses to analyze any interaction effects. The analysis was performed via the MANOVA() function, and the post hoc pairwise comparisons were conducted via the EMMEANS() function from the “bruceR” package in R^52^.

We employed a commonly used algorithm to calculate the D_600_ score^43^ to reflect participants’ implicit attitudes (see Supplementary Note 9). A paired-sample *t*-test was conducted to compare participants’ implicit attitudes before and after sleep, using the TTEST() function from the “bruceR” package in R^52^.

The differences in selecting the cued name and uncued name as allocators were analyzed via the chi-square test with the chisq.test() function from the “stats” package in R.

We conducted paired-sample *t*-tests via the TTEST() function to compare participants’ explicit degree of likeability and social willingness for the cued and uncued names.

## Supplementary information

Supplementary Information

## Data Availability

All preprocessed and aggregated data, as well as the code relevant for reproducing the statistical tests presented in the article, are available on the Open Science Framework (https://osf.io/8nhv6/). Raw data can be obtained from the corresponding author upon request.

## Code availability

The code required for data processing is available on the Open Science Framework (https://osf.io/8nhv6/).
