# Supplementary material for: Updating social evaluation during sleep
Source: NPJ Sci Learn. 2025 Aug 30;10:65. doi: 10.1038/s41539-025-00356-9 (PMC12398527; doi:10.1038/s41539-025-00356-9)
Supplement: Supplementary file 1 — Supplementary Information [file 41539_2025_356_MOESM1_ESM.pdf]

Supplementary Materials for

Updating Social Evaluation During Sleep

Haoyun Zhao,[1, #] Xiao Lin,[2, #] Kai Yuan,[2] Xiaoqing Hu,[3] Xikai Wang,[1] Waxun Su, [4] Qiandong Wang,[4, *] Lin Lu,[1,2, 5,*]

[1] Peking-Tsinghua Center for Life Sciences and Academy for Advanced Interdisciplinary Studies, Peking University, Beijing 100871, China

[2] Peking University Sixth Hospital, Peking University Institute of Mental Health, NHC Key Laboratory of Mental Health (Peking University), National Clinical Research Center for Mental Disorders (Peking University Sixth Hospital), Beijing 100191, China

[3] Department of Psychology, The State Key Laboratory of Brain and Cognitive Sciences, The University of Hong Kong, Hong Kong SAR, China

[4] Beijing Key Laboratory of Applied Experimental Psychology, National Demonstration Center for Experimental Psychology Education (Beijing Normal University), Faculty of Psychology, Beijing Normal University, Beijing 100875, China

[5] PKU-IDG/McGovern Institute for Brain Research, Peking University, Beijing 100871, China.

# These authors contributed equally to this work

* Corresponding authors

Prof. Lin Lu, M.D., Ph.D.

Institute of Mental Health/Peking University Sixth Hospital, 51 Huayuanbei Road, Haidian District, Beijing, 100191, China

Tel: +86-10-82805308

E-mail: linlu@bjmu.edu.cn

Dr. Qiandong Wang, Ph.D.

Faculty of Psychology, Beijing Normal University, 19 Xinjiekouwai Avenue, Haidian District, Beijing 100875, China

E-mail: wangqd@bnu.edu.cn

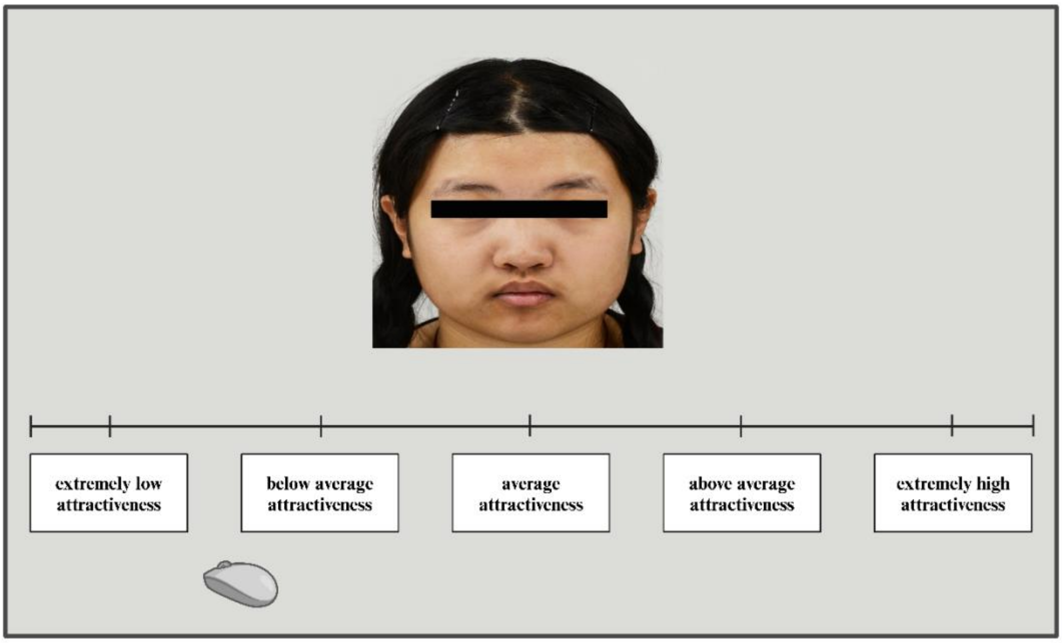

Supplementary Figure 1. Facial attractiveness online rating task for selecting facial stimuli. Participants judged the facial attractiveness of different faces based on a scale of 1 to 5 (1: extremely low attractiveness; 2: below average attractiveness; 3: average attractiveness; 4: above average attractiveness; 5: extremely high attractiveness). ***Note.*** **The eyes of the real face photos are covered to protect privacy. The model represented in the figure was obtained from the Tsinghua University Facial Expression Database [1], and the original database confirms that written informed consent was obtained from all face models for the use of their images in scientific research, including publications.**

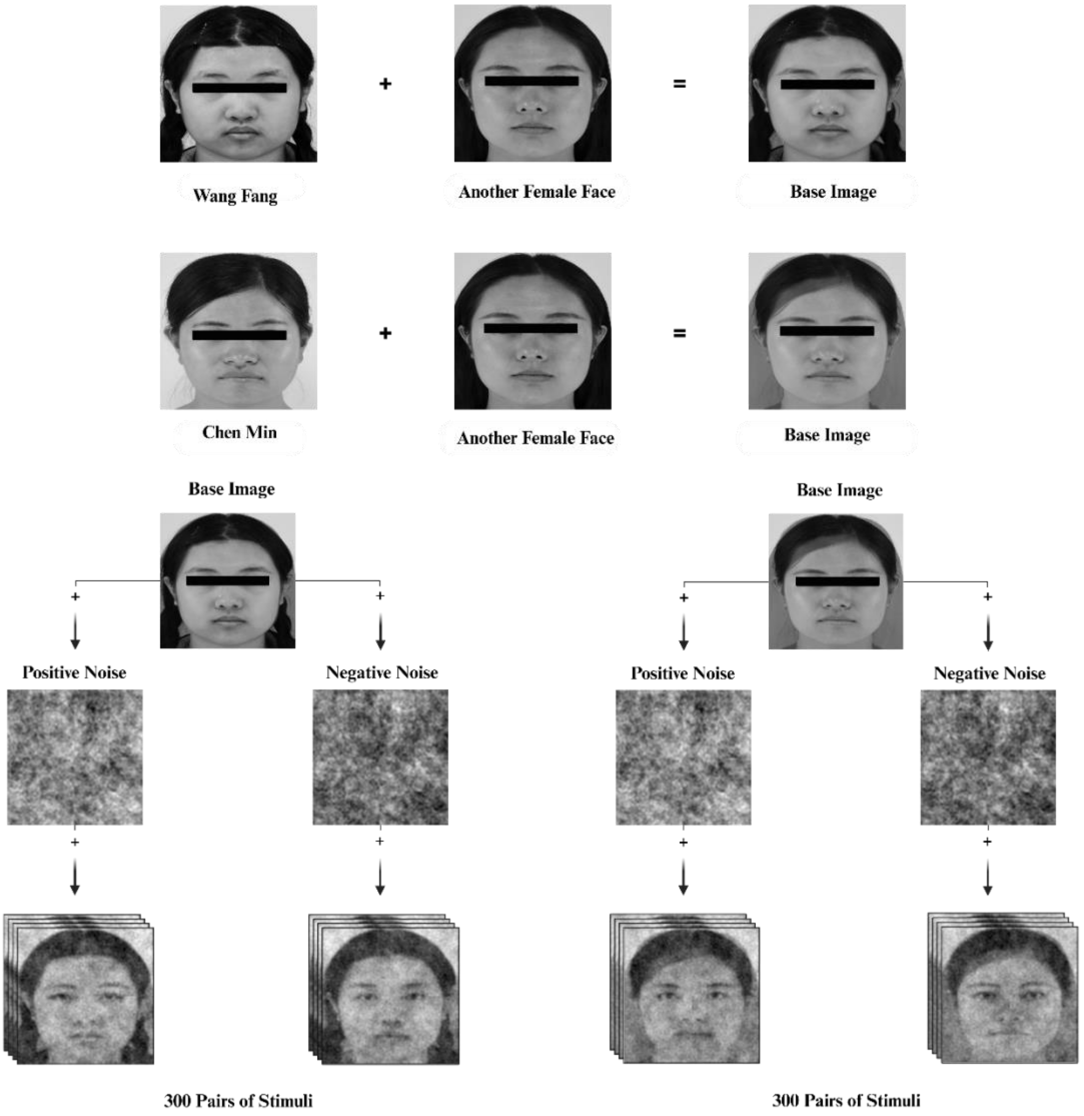

Supplementary Figure 2. Noise-infused stimuli images in the reverse correlation image classification task. The pixel dimensions of the two facial images used in the encoding phase were both standardized to 512 x 512 pixels. Each of the two facial images was morphed separately with another female face sourced from the Tsinghua University Facial Expression Database [1]. The morphed images were then subjected to blurring effect. Positive and negative noise was added to the blurred images. Images were paired: one with positive noise and the other with negative noise, resulting in 300 image pairs for each of the two potential cued face targets.

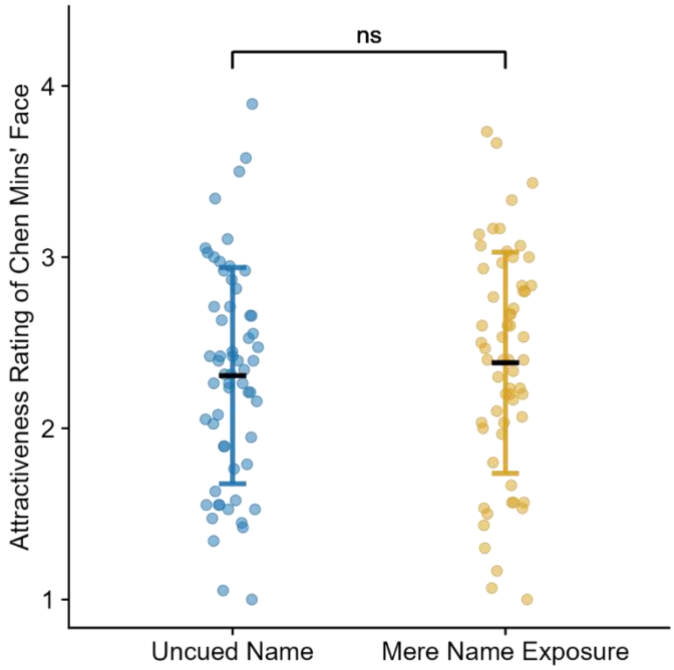

Supplementary Figure 3. Attractiveness of mental face representations of the uncued (Chen Min's) face between the mere exposure group and the experimental group.

Attractiveness ratings were modeled with the following fixed effects: rate ~ conditions + (1|subID)+(1|expID). Here, the variable 'conditions' refers to the uncued face in the mere exposure group and in the experimental group. Participant's ID for the sleep experiment (expID) was included as a random intercept to account for individual variability in representation generation, while participant's ID for the ratings (subID) was included as a random intercept to account for individual variability in rating. No significant difference was found in the attractiveness of mental face representations of the uncued name between the mere exposure group and the experimental group.

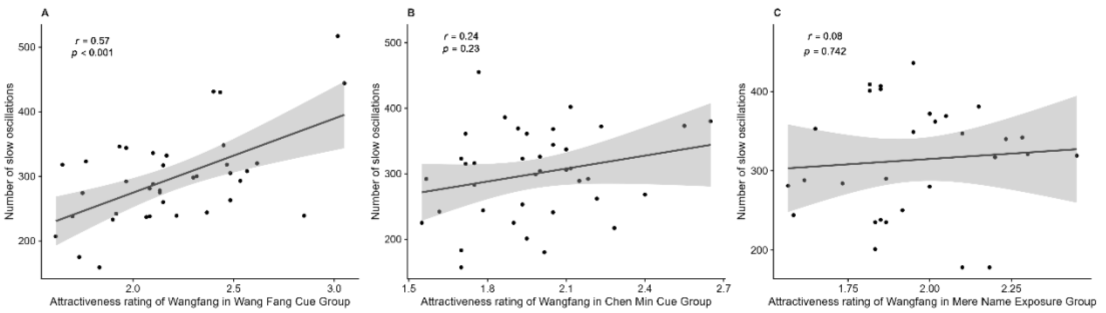

Supplementary Figure 4. The correlation between the number of slow oscillations and the attractiveness perceptions towards mental facilal representation of Wang Fang, across three groups: Wang Fang Cue Group, Chen Min Cue Group, and Mere Name Exposure Group. (A) The correlation between the number of slow oscillations and perceived attractiveness of Wang Fang within the Wang Fang Cue group. (B). The correlation between the number of slow oscillations and perceived attractiveness of Wang Fang within the Chen Min Cue group. (C). The correlation between the number of slow oscillations and perceived attractiveness of Wang Fang within the Mere Name Exposure group.

Supplementary Table 1. Traits and corresponding ratings for the two face names.

Face Names	Positive/ Negative	Traits	Average Valence	Average Arousal	Average Familiarity	Average Meaningfulness
Wang Fang	Positive	开明 (Open-minded)	5.67	6.06	5.97	4.64
Wang Fang	Negative	邋遢 (Sloppy)	2.15	6.44	6.03	4.91
Wang Fang	Positive	进取 (Ambitious)	5.67	6.25	6.1	5
Wang Fang	Negative	盲从 (Uncritical)	2.48	5.44	5.83	4.61
Wang Fang	Positive	乐观 (Optimistic)	5.55	6.38	6.3	5.42
Wang Fang	Negative	肤浅 (Superficial)	2.48	6.09	6.1	4.67
Wang Fang	Positive	敏锐 (Acute)	5.55	6.28	5.83	4.61
Wang Fang	Negative	庸俗 (Vulgar)	2.27	5.91	6.27	4.73
Wang Fang	Positive	热情 (Passionate)	5.73	6.22	6.37	4.79
Wang Fang	Negative	软弱 (Feeble)	2.58	6.41	6.1	4.58
Chen Min	Positive	敬业 (Dedicated)	5.97	6.19	6.3	5.27
Chen Min	Negative	愚昧 (Ignorant)	2.24	6.41	6.27	4.61
Chen Min	Positive	坚强 (Tenacious)	5.7	6.22	6.3	4.85
Chen Min	Negative	猖狂 (Frenized)	2.39	5.56	5.93	4.52
Chen Min	Positive	风趣 (Witty)	5.76	5.91	6	4.97
Chen Min	Negative	轻浮 (Frivolous)	2.45	5.66	6.17	5
Chen Min	Positive	刻苦 (Hard-working)	5.64	6.25	5.9	4.79
Chen Min	Negative	急躁 (Impatient)	2.55	5.78	6	4.76
Chen Min	Positive	勇敢 (Courageous)	5.79	6.31	6.17	4.61

Chen Min	Negative	无能 (Inept)	2.3	6.13	6.27	4.79

Supplementary Table 2. Aggregate ratings of traits for the two face names.

	Wang Fang Traits	Chen Min Traits
Aggregate Valence	40.13	40.79
Aggregate Arousal	61.48	60.42
Aggregate Familiarity	60.9	61.31
Aggregate Meaningfulness	47.96	48.17

Supplementary Table 3. Concept and attribute words used in the IAT.

Concept words	Attribute words
Positive Traits	Kind，Cute，Safe，Gentle，Honest，Wealthy，Happy， Amiable
Negative Traits	Clumsy，Terrible，Weird，Dangerous，Ugly， Poor， Sad， Helpless
Wang Fang	Name derivations (WangFang, Xiao Fang, A Fang, Wang Fang)
Chen Min	Name derivations (ChenMin, Xiao Min, A Min, Chen Min)

Supplementary Table 4. 16 Positive words we used during sleep cue exposure

Words	Average Familiarity
真诚 (Sincere)	6.53
聪明 (Intelligent)	6.53
诚实 (Honest)	6.53
友善 (Friendly)	6.5
耐心 (Patient)	6.47
冷静 (Calm)	6.47
勤奋 (Diligent)	6.47
公正 (Fair)	6.41
细心 (Attentive)	6.41
自信 (Confident)	6.38
认真 (Conscientious)	6.38
谦虚 (Humble)	6.38
乐观 (Optimistic)	6.38
幽默 (Humorous)	6.38
可靠 (Trustworthy)	6.34
主动 (Proactive)	6.34

Supplementary Table 5. Round number, key response, and trial numbers in the IAT.

Round	A_key	L_key	Trial Numbers
1	Positive Traits	Negative Traits	16
2	Wang Fang	Chen Min	16
3	Wang Fang or Positive Traits	Chen Min or Negative Traits	24
4	Wang Fang or Positive Traits	Chen Min or Negative Traits	48
5	Negative Traits	Positive Traits	16
6	Wang Fang or Negative Traits	Chen Min or Positive Traits	24
7	Wang Fang or Negative Traits	Chen Min or Positive Traits	48

Supplementary Table 6. Average duration and number of cues played.

	Wang Fang Cue Group	Chen Min Cue Group	Mere Name Exposure Group	*F*	*P*-value
Average Duration of Cue Played (seconds, (mean ± *SD*)	739.1 ± 470.2	802.7 ± 432.3	946.5 ± 392.4	1.89	0.16
Average Number of Cue Played (count, mean ± *SD*)	370.0 ± 235.7	394.5 ± 212.5	474.2 ± 196.7	2.00	0.14

Supplementary Note 1. We recruited 60 native Chinese participants from the Naodao online testing platform (https://www.naodao.com/) (a validated online experiment platform) to complete the facial attractiveness rating task in which they judged the facial attractiveness of different faces based on a scale of 1 to 5 (1: extremely low attractiveness; 2: below average attractiveness; 3: average attractiveness; 4: above average attractiveness; 5: extremely high attractiveness) (see **Supplementary Figure 1**). All face stimuli portraying neutral expressions were selected from the Tsinghua-FED (Tsinghua facial expression database), a validated facial expression database comprising high-quality color photographs. All facial stimuli were 1500 x 2000 pixels, and those with head tilt were corrected using Adobe Photoshop CC 2017.

In the rating task, 32 female faces from Tsinghua-FED were rated on attractiveness. All stimuli were resized to 0.5 of their original size and presented in the center of the screen. The order of the facial stimuli presented was randomized among participants. In total, responses of 56 online raters (28 females, 28 males) within the age range of 18-35 were analyzed, and four participants who self-indicated to be distracted during the rating task or attributed the same rating to all the face stimuli on 95% rating trials were excluded from further analysis.

For the formal experiment, we selected two female faces with the lowest attractiveness rated by 56 raters as our test stimuli. The average attractiveness ratings of female face1 and female face2 were 1.61 (SD = 0.56) and 1.63 (SD = 0.59), respectively. No significant differences in the attractive ratings were found between the two faces, $t(55) = 0.20$, $p = .84$.

Supplementary Note 2. The facial stimuli used in the reverse correlation image classification (RCIC) task underwent the following image processing techniques used in prior research [2-4]: (i) The

pixel dimensions of the two facial images used in the encoding phase were both standardized to 512 x 512 pixels; (ii) Each of the two facial images was morphed separately with another female face (also at a resolution of 512 x 512 pixels) sourced from the Tsinghua University Facial Expression Database (software: WinMorph 3.01; warping: moderate; frame number: 21); (iii) The morphed images were then subjected to blurring effect; (iv) Positive and negative noise was added to the blurred images. Images were paired: one with positive noise and the other with negative noise, resulting in 300 image pairs for each of the two potential cued face targets (see **Supplementary Figure 2**).

Supplementary Note 3. Personality traits were derived from **Zhang et al., (2018)** [5] and were evaluated on valence, arousal, familiarity, and meaningfulness. The mean ratings are shown in Table S1. For Wang Fang, the positive traits assigned were "ambitious", "acute", "optimistic", "passionate", and "open-minded", while the negative traits were "sloppy", "feeble", "vulgar", "uncritical", and "superficial". Chen Min's positive traits included "witty", "courageous", "dedicated", "tenacious", and "hard-working", with her negative traits being "ignorant", "impatient", "frivolous", "inept", and "frenzied".

We balanced the attributes of the two face names across multiple dimensions **(Supplementary Table 2).** For Wang Fang, the aggregate scores of the allocated personality traits for valence, arousal, familiarity, and meaningfulness were 40.13, 61.48, 60.9, and 47.96, respectively. For Chen Min, the aggregate scores of the allocated personality traits were 40.79 for valence, 60.42 for arousal, 61.31 for familiarity, and 48.17 for meaningfulness.

Supplementary Note 4. All semantic-associative acoustic cues were generated using an AI voice-

over software with uniform parameters (software name: Voice-over Pocket, Speed: -50, Tone: -5, Emotion: Universal). Acoustic cues were initiated once the participants were confirmed to be in the slow-wave sleep phase, as monitored by PSG (polysomnography).

Supplementary Note 5. The words used in the IAT were divided into four categories: Positive Traits, Negative Traits, and two name categories - Wang Fang and Chen Min (see **Supplementary Table 3**). The concept and attribute words form the basis of the IAT's design, which are strategically chosen to elicit automatic responses that reveal underlying biases and associations. The categorization and pairing of these words in different rounds of the IAT enable a comprehensive analysis of implicit attitudes, providing valuable insights into how individuals subconsciously link certain traits with specific names.

The IAT was divided into seven rounds, each with a unique focus and a predetermined number of trials (see **Supplementary Table 5**). The first round consisted of 16 trials where participants were asked to categorize traits as 'Positive' or 'Negative'. This establishes a fundamental understanding of how individuals differentiate between generally positive and negative characteristics.

The second round, also comprising 16 trials, shifts the focus to identifying specific names – "Wang Fang" and "Chen Min". This helps in assessing the participant's ability to recognize and categorize these names, which is essential for the more complex tasks in the subsequent rounds.

In the third round, which includes 24 trials, the test becomes more intricate as it combines the elements of the previous two rounds. Participants were required to categorize items as either "Wang Fang or Positive Traits" and "Chen Min or Negative Traits". This round is crucial as it begins to assess the automatic associations between the given names and the traits, providing insights into

whether the names “Wang Fang” and “Chen Min” are more readily linked with positive or negative connotations in the participant’s mind.

The fourth round is an extension of the third, but with an increased number of trials, totaling 48 trials. The additional trials in this round aim to enhance the reliability of the measurements, allowing for a more accurate assessment of the participant’s implicit associations.

Subsequently, the fifth round, with 16 trials, reverses the categories from the first round, asking participants to categorize traits as “Negative Traits” or “Positive Traits”. This reversal is significant for analyzing the impact of changing the associations and controlling potential biases in response times related to the positioning of the categories.

The sixth round, similar to the third but with reversed pairings, includes 24 trials where items are categorized as “Wang Fang or Negative Traits” and “Chen Min or Positive Traits”. This round further explores the strength and nature of the associations between the names and the traits, but in a reversed context.

Finally, the seventh round repeats the structure of the sixth round but doubles the number of trials to 48.

Supplementary Note 6. To minimize sound interference with the participants’ sleep, the speakers were positioned 1.5 meters away from the beds, ensuring that the sound level reached the participants’ bedside was approximately 50 decibels.

Supplementary Note 7. The acoustic cues were played using the Psychtoolbox in the MATLAB 2020b program. The program automatically recorded the start time of each acoustic cue and sent

markers to the polysomnography (PSG) recordings to synchronize them with the MATLAB program's timeline. To ensure consistency in the total exposure duration across different groups, we implemented a specific adjustment method for the semantic-associative acoustic cues. For example, with cues such as "Wang Fang + trustworthy", we deducted the duration of the name "Wang Fang" from the total duration, isolating the duration of the word "trustworthy". This process was applied to all 16 positive traits, determining their individual durations, and then calculating an average duration for these adjectives. This average duration was then added as a silent period following the name in the name-only exposure group. Thus, after the name "Wang Fang" is pronounced, there is a silent period equal to the average duration of the adjectives, ensuring that the total duration of acoustic cue exposure remains roughly equivalent across all groups.

After the task was finished, the program automatically recorded the time elapsed since the initial tagging before the first acoustic cue (e.g., 100.54 seconds) and the count of each acoustic cue played. Additionally, the experimenter could press the space bar to initiate the acoustic cue playback and press it again to pause it. Upon the completion of the sleep, for each participant, one cue exposure file including the total duration of cue played and the number of total cue played was automatically recorded and saved. In addition, there is no statistically significant differences in average duration of cue played or average number of cued played across the three groups (see Supplementary Table 6).

Supplementary Note 8. The memory bias was calculated for both pre-sleep and post-sleep memory bias tests, with the following formulas:

$$(1)\ \text{Positive memory bias for Wang Fang} =$$

$$\frac{\text{Number of correct answers for positive traits of Wang Fang}}{\text{Total number of positive traits of Wang Fang answered}}$$

(2) Negative memory bias for Wang Fang =

$$\frac{\text{Number of correct answers for negative traits of Wang Fang}}{\text{Total number of negative traits of Wang Fang answered}}$$

(3) Positive memory bias for Chen Min =

$$\frac{\text{Number of correct answers for positive traits of Chen Min}}{\text{Total number of positive traits of Chen Min answered}}$$

(4) Negative memory bias for Chen Min =

$$\frac{\text{Number of correct answers for negative traits of Chen Min}}{\text{Total number of negative traits of Chen Min answered}}$$

Supplementary Note 9. To quantify the implicit attitude, we employed a commonly used algorithm[6] from the literature to calculate the D600 score for each participant's pre- and post-sleep IAT data. The calculation process was as follows:

(1) Exclude all trials with response times shorter than 300ms or longer than 3000ms.

(2) Calculate the average response time for correct trials in rounds 3 and 4, and for rounds 6 and 7.

(3) Determine the standard deviation of response times for correct trials in rounds 3, 4, 6, and 7.

(4) Add 600ms to the response times for incorrect trials in rounds 3, 4, 6, and 7.

(5) Recalculate the average response times for rounds 3 and 4, and for rounds 6 and 7, using the updated data from step 4.

(6) Calculate the difference between the average response times for rounds 6 and 7 and for rounds 3 and 4, using the updated data from step 5, then divide this difference by the standard deviation from step 3 to obtain the D600 score.

Supplementary References

1 Yang, T. *et al.* Tsinghua facial expression database–A database of facial expressions in Chinese young and older women and men: Development and validation. *PloS one* **15**, e0231304 (2020).

2 Dotsch, R. & Todorov, A. Reverse correlating social face perception. *Soc. Psychol. Pers. Sci.* **3**, 562-571 (2012).

3 Todorov, A., Olivola, C. Y., Dotsch, R. & Mende-Siedlecki, P. Social attributions from faces: Determinants, consequences, accuracy, and functional significance. *Annu. Rev. Psychol.* **66**, 519-545 (2015).

4 Oh, D., Dotsch, R. & Todorov, A. Contributions of shape and reflectance information to social judgments from faces. *Vision Res* **165**, 131-142 (2019).

5 Zhang, Y., Pan, Z., Li, K. & Guo, Y. Self-serving bias in memories. *Exp. Psychol.* **65**, 236-244 (2018).

6 Greenwald, A. G., Nosek, B. A. & Banaji, M. R. Understanding and using the implicit association test: I. An improved scoring algorithm. *J. Pers. Soc. Psychol.* **85**, 197 (2003).
